# Multi Scale Analysis of the Retting and Process Effect on the Properties of Flax Bio-Based Composites

**DOI:** 10.3390/polym15112531

**Published:** 2023-05-31

**Authors:** Mohamed Ragoubi, Morgan Lecoublet, Medhi Khennache, Christophe Poilane, Nathalie Leblanc

**Affiliations:** 1 UniLaSalle, Unité de Recherche Transformation et Agro-Ressources, VAM²IN (ULR 7519 UniLaSalle—Université d’Artois), 76130 Mont-Saint-Aignan, France; mkhennache@hotmail.fr (M.K.); nathalie.leblanc@unilasalle.fr (N.L.); 2 Normandie University, ENSICAEN, UNICAEN, CEA, CNRS, CIMAP, 14000 Caen, France; christophe.poilane@unicaen.fr

**Keywords:** flax morphology, retting effect, processing parameters, property, physical and mechanical properties, bio-based materials, porosity

## Abstract

This research aimed to evaluate, at different scales (technical flax fiber, fiber band and flax composites, bio-based composites), the effect of retting and processing parameters on the biochemical, microstructural, and mechanical properties of flax-epoxy bio-based materials. On the technical flax fiber scale, a biochemical alteration of the fiber was observed as the retting increased (a decrease of the soluble fraction from 10.4 ± 0.2 to 4.5 ± 1.2% and an increase of the holocellulose fractions). This finding was associated with the degradation of the middle lamella, favoring the individualization of the flax fibers observed at retting (+). A direct link was established between the biochemical alteration of technical flax fibers and their associated mechanical properties (decrease of the ultimate modulus 69.9 to 43.6 GPa and maximum stress from 702 to 328 MPa). On the flax band scale, the mechanical properties are driven by the interface quality between the technical fibers. The highest maximum stresses were reached at level retting (0) with 26.68 MPa, which is lower compared to technical fiber. On the bio-based composites scale, setup 3 (T = 160 °C) and the high retting level (+) are the most relevant for a better mechanical response of flax bio-based materials.

## 1. Introduction

Nowadays, natural fiber composites are highly recommended in many engineering applications for different industrial sectors due to their technical and eco-friendly properties [1,2,3]. The natural reinforcements come from different origins and are grouped into families according to their role in the plant. Some, such as flax or jute fibers, are found in the stem of the plant, while others, such as kapok or cotton, are contained in the seed [4]. The conditions of culture, growth, and arrangement in the plant will govern their morphological, structural, physicochemical, and mechanical properties [4]. A plant fiber such as flax has a complex microstructure based on multiple cell layers. As described by Keryvin et al., 2015, the external cell wall is a 0.2 µm thick layer named the primary cell wall [5]. The major cell wall is the secondary wall, divided into three different layers—S1 (0.5–2 µm thick), S2 (5–10 µm) and S3 (0.5–1 µm). This wall can provide the reinforcement of the plant structure. It is composed of 4 main biochemical fractions: cellulose, hemicellulose, lignin, and extractive compounds [6,7,8,9,10]. Cellulose is the main component of the S2 layer and is present in both amorphous and crystalline arrangement [11]. Microfibrils of crystalline cellulose are responsible for the mechanical properties of the fiber [11,12]. The analysis of different articles shows great variations in the biochemical composition [6,7,13,14]. The cellulose content is between 49 and 85%, hemicellulose from 9 to 29%, lignin from 2 to 18%. The reason for such variation may come from many factors, such as the method of measurement, the flax variety, and the applied retting. Moreover, retting is a natural process involving the degradation of pectins present in the flax stem. This process will allow the fiber bundles, naturally present at the periphery of the stem, to be separated into technical fibers [15]. For flax and hemp fibers, the traditional retting processes are water retting and dew retting; both are based on degradation by micro-organisms. A recent research paper [13] demonstrated that, in the L × a × b × spectrocolorimetric coordinates, the retting affects the color of the fiber, gradually making the fiber darker. A significant impact on the mechanical properties has been reported elsewhere [8,12,13,15]. However, other methods are being studied to better master the retting control, such as microwave assisted retting and enzymatic assisted retting [16]. The retting and, consequently, the fiber individualization will have a significant impact on many properties. Lecoublet et al., 2021, found an increase in cellulose content and a decrease in hemicellulose and extractive substance content in flax [13]. The crystallinity of the fibers is directly related to the cellulose content, and the crystallinity of flax fibers tends to increase with retting [13,17]. However, the mechanical properties of technical flax fibers tend to decrease, mainly due to the decrease in the quality of the fiber/fiber interface, which no longer plays the role of force transfer.

The advantages of plant fiber biocomposites lie in their versatility. Due to the plethora of fibers and matrices, the desired properties (mechanical, morphological, thermal) can be optimally adjusted according to the intended use. The relative low density of plant fibers allows specific properties to be obtained, making them desirable for applications requiring lightweight products [1,13,18,19] such as automobile, aeronautics and sport. Deng and Tian, 2015, showed that using French flax fibers, instead of glass fibers, in mat structure with a polypropylene matrix, results in a global decrease of environmental impacts [20]. They can be locally produced, which limits the impact of transporting the raw materials to the manufacturing site. Due to the multiscale structure of plant fibers [5], special care must be taken during fiber preparation, otherwise it can lead to critical problems in bio-based composite materials, such as poor wettability, thermal damage to the fiber, degradation at the fiber/matrix interface, and high porosity. The processing has a crucial impact both on the curing efficiency and on the mechanical performances of the final materials. Granado et al., 2018, shows that a shorter processing time results in a lower conversion rate of the epoxy resin [21]. Additionally, the curing temperature positively impacts the conversion reaction [22]. They showed that the tensile modulus varied positively with increasing processing temperature, increasing the monomer conversion rate and thus the charge transfer quality of the matrix. They also mention the negative impact of a too-high processing temperature, degrading the fibers. The manufacturing process can itself be a source of defects. Regarding the impact of retting on the bio-based composites, Martin et al., 2013, showed that the Young modulus and the tensile strength of polypropylene (PP):short flax fiber composites were positively influenced by retting [8]. They associated this with easier splitting of the fiber bundles during processing, resulting in smaller fibers and a larger interface. Using Polypropylene-graft-maleic anhydride (PP-g-MA):flax composites, Chabbert et al., 2020, also observed a small but significant improvement in the Young’s modulus of bio-based composites with retting [12]. They also associated this improvement with an enhancement of the interactions between the fiber and the matrix. Therefore, it is necessary to have a complete knowledge of the different components to favor the integration of bio-based materials. For applications with high vibrational stresses, the viscoelastic properties of the bio-based composites produced must be fully understood [23,24,25,26]. Muralidhar, 2013, studied the viscoelastic properties of flax-reinforced epoxy composites and showed that the tensile properties appeared to be positively influenced by the fiber volume fraction, matrix properties, and fiber/matrix bonding [26]. Compared to neat epoxy, flax composites with a W_f_ = 31% showed a 75% increase of the storage modulus at room temperature. However, Oksman et al., 2003, observed a 200% increase of the storage modulus (at room temperature) for flax composite compared to polylactic acid (PLA) matrix [25].

This research aimed to evaluate, at different scales (technical flax fiber, fiber band, and bio-based materials), the effect of retting and processing parameters on the biochemical, microstructural, and mechanical properties of flax-epoxy bio-based materials. The originality of this work lies in the analysis of the influence of the retting and the processing on a large scale of the material, from the unitary fiber to the biobased composite. This in-depth analysis will allow us to propose an optimal bio-based composite. Although the effect of retting has been widely studied, the originality of our work relies on the different combined parameters linked both to retting level, and processing parameters to achieve the best performances of flax bio-based materials. It provided additional answers to the effect of retting and processing parameters at a different materials scale. The biochemical, microstructural, mechanical, and viscoelastic properties of flax-based composites have been studied in depth. Our results provided a great help to our industrial partner to select better conditions (retting and process) for flax reinforcements and optimize the whole properties of bio-based flax composites, while saving time and money on the manufacturing materials step with a more suitable process.

## 2. Materials and Methods

### 2.1. Raw Materials

Epoxy XB3513 resin and Aradur 5021 polyimide crosslinker were used as the matrix polymer. The weight ratio (Aradur crosslinker to the epoxy) was about 24%. It was provided by VITECH COMPOSITES, Sainte-Maure-de-Touraine, France. For the plant fiber, flax of the Bolchoi variety was used. The flax was grown in Romilly La Puthenay, Normandy, France. The seeding, harvesting, and retting conditions are summarized in Table 1. The retting dates were set at August 7th, 2017 (W1), August 28th, 2017 (W2), and September 22nd, 2017 (W3). The latter date was chosen according to the cumulative degrees received by the plants, 1014 °C, which gave the maturity levels of fibers. Winding dates were based on visual estimation of retting level and according to climatic conditions. More details were reported in our previous paper [13]. The flax has been scutched, but no hackling step, nor thermal, chemical, or physical preparation was applied.

### 2.2. Preparation of Bio-Based Composites

Once the flax fibers have been prepared, they were arranged into a veil band of unidirectional-oriented flax fiber and glued with an agent spray. The flax veils were impregnated with the epoxy resin to form prepregs sheets. The impregnation step was carried out on non-woven UD flax bands with a 110 g/m^−2^ grammage. The water-diluted epoxy was then sprayed on the flax bands and, finally, pre-cured at 120 °C. The resulting flax-epoxy prepregs presented a fiber content of Wf = 50% ± 3.

Three different processing protocols were proposed, called Setup 1, Setup 2 and Setup 3. Table 2 describes the different steps and details of the processing protocols. For information, Setup 1 was a processing protocol proposed by the industrial partner. Setups 2 and 3 were protocols considered for this study to optimize their technical parameters. For the elaboration step, flax-epoxy prepregs were cut into 30 cm squares and hot-pressed with a Scamex 20 T 300 × 300 press (Scamex, Isques, France), performed at Unilasalle Rouen (Mont-Saint-Aignan, France). Then they were stacked in a UD pattern and placed between two metal plates. Two sheets of PTFE were added to facilitate the demolding step. At the end, we obtained bio-based composites plates with an average thickness of 493 ± 31 µm and average density of 1.262 ± 0.024 g·cm^−3^.

### 2.3. Analysis Methods of Bio-Based Materials

#### 2.3.1. Biochemical Analysis

The chemical composition (cellulose, hemicelluloses, and lignin) on the flax samples were performed according to the AFNOR standard (XPU44-162). The tests were carried out on 1 g of material, with three repetitions for each sample, using a FOSS manufactured raw fiber extractor device from FischerScientific (Waltham, MA, USA) and carried out at Unilasalle Rouen (Mont-Saint-Aignan, France). This method allows us to determine the biochemical content by fractionation of plant matter using different solvents. The results for the different chemical parameters were expressed in relation to the dry matter. The analytical dry matter was measured in a ventilated oven at 105 °C for 48 h. Cellulose, hemicelluloses, and lignin were determined from the neutral detergent insoluble residue (NDF), acid detergent insoluble residue (ADF), and acid detergent and H_2_SO_4_ (%) (*w*/*w*) insoluble residue.

#### 2.3.2. Crystallinity Index

The crystallinity index of flax fibers was calculated with the XRD technique according to the method of Segal et al., 1959 [27]. Flax fibers are compressed as a disk (thickness of 3 mm and 30 mm for diameter). X-ray diffractograms were recorded from 2θ = 3 to 60°, with a scan rate of 0.04°·s^−1^. The crystallinity index (CI (%)) is calculated from the following equation, Equation (1) [27]:

(1) $$CI\;\left(\%\right)=\frac{I_{002}\times I_{am}}{I_{002}}\times 100$$

where *CI* (%) is the relative degree of crystallinity, *I*_002_ is the maximum intensity of the 002-lattice diffraction, and *I_am_* is the intensity of diffraction of the amorphous material at 2θ = 18°.

#### 2.3.3. Density Analysis

The density of flax fibers was performed with an AccuPyc 1330 Pycnometer from Micromeritics, Norcross, GA, USA, and carried out at Unilasalle Rouen (Mont-Saint-Aignan, France), using a 20 cc sample cell under argon flow. Three tests were carried out on each flax sample and each result is an average of three measurements.

#### 2.3.4. Tensile Test for Technical Flax Fibers

Mechanical analysis of technical flax fibers was carried out by using an MTS Criterion (MTS, New Baltimore, MI, USA) 43 tensile machine associated with a 0.5 kN load cell. Tested lengths ranged from 14 to 100 mm, with 2 mm increments (thirty-three samples by retting mode). Displacement rate was set at 1 mm·min^−1^. The measurements of the cross section of technical fibers were based on a densiometry method and reported with more details as proposed in our previous article [14].

#### 2.3.5. Tensile Test for Scutched Flax Band

**Figure 1:**
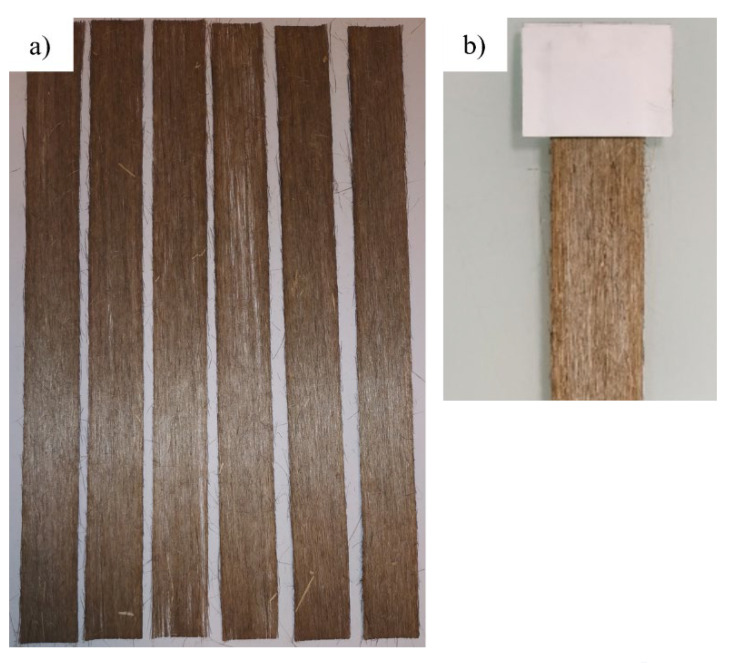
(**a**) Scutched flax specimens, (**b**) Zoom on the grip section.

Flax bands, i.e., flax veil (density = 110 g·m^−2^) were laser cut into 285 × 20 mm strips with a Mllaser ML-W1290 laser cutter (MLLASER, Pont-à-Mousson, France) (Figure 1a). To prevent the reinforcement from burning, the latter is sandwiched between two sheets of paper. After that, 40 × 25 mm paper sheets are glued to the ends of the materials, as shown in Figure 1b. The mechanical tests are carried out on a Shimadzu traction machine (Shimadzu Corp, Kioto, Japan) with a 200 N capacity load, displacement rate of 2 mm·min^−1^, and laboratory conditions of 23 °C and 65% humidity. Due to the nature of the tested material, no extensometer has been installed. The gauge length is then 250 mm. For the stress determination, the cross section is determined by the mass of the specimen and the density of the flax, according to the Equation (2):

(2) $$cross\;section=\frac{sample\;weight\times 1000}{flax\;density\times sample\;length}\;$$

where the *cross section* is expressed in mm^2^, *sample weight* is expressed in g, the *flax density* in g·mm^−3^ and the *sample length* is expressed in mm.

#### 2.3.6. Tensile Test for Bio-Based Composite Materials

**Figure 2:**
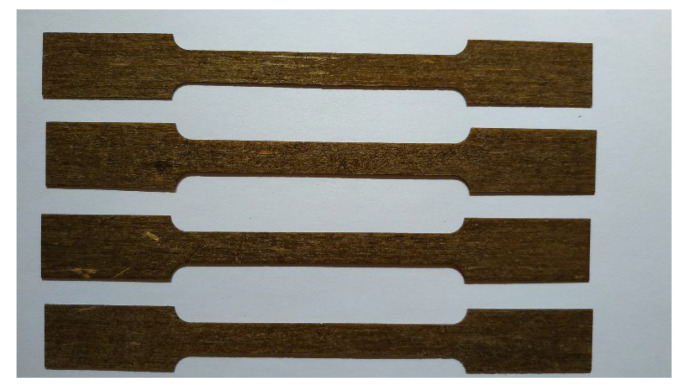
Flax bio-based samples for tensile test.

Mechanical analysis of bio-based composite was carried out on a Shimadzu traction machine with a 50 kN capacity load, displacement rate of 2 mm·min^−1^, with laboratory conditions of 23 °C and 65% relative humidity, and conducted at Unilasalle Rouen (Mont-Saint-Aignan, France). Tensile specimens were prepared according to ISO 527 (Figure 2) and cut with a Mllaser ML-W1290.

#### 2.3.7. Dynamic Mechanical Analysis

Dynamic mechanical analysis (DMA) was carried out in 3-point bending in a Netzsch DMA 242 system and carried out at Unilasalle Rouen (Mont-Saint-Aignan, France). Samples dimensions are 40 mm × 10 mm × 0.5 mm. The heating setup consists of a temperature slope from 30 to 130 °C at 3 °C·min^−1^. During this analysis, the oscillation is strain-controlled, with a dynamic amplitude up to 30 µm and a force range between 4 ± 4 N. Measurements were carried out from 0.1 to 1 Hz frequencies.

#### 2.3.8. SEM Analysis

SEM analysis was performed with a JEOL-JSM100 Scanning Electron Microscope. The tests were conducted under 15 kV with magnification ranging from ×37 to ×200. No coating was applied to either material. Three specimens were examined for each material, placed on a carbon adhesive tape to observe the fibers and the bio-based materials in the transverse axis.

## 3. Results and Discussions

### 3.1. Biochemical and Microstructural Analysis of Flax Fiber

**Figure 3:**
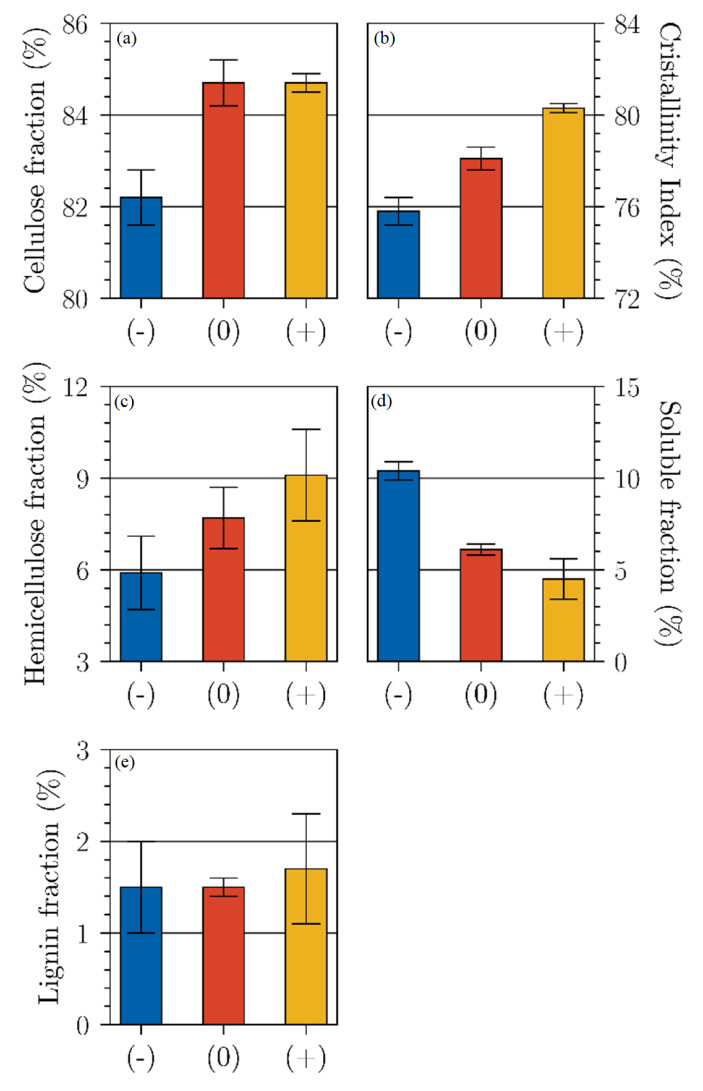
Biochemical analysis of flax fibers versus the retting applied (**a**) Cellulose fraction, (**b**) Crystallinity Index, (**c**) Hemicellulose fraction, (**d**) Soluble fraction, (**e**) Lignin fraction. (Error bars represent the standard deviation).

Figure 3 shows the biochemical analysis of the different retted flax. The cellulose fraction varies from 82.2 to 84.7% between the (−) and (+) retting. The hemicellulose fraction tends to increase continuously (varying from 5.9 to 9.1%) with the retting level. The lignin fraction remains constant at 1.5% and the soluble fraction (made of pectins, proteins, tannins) decreases steadily from 10.4 to 4.5%, respectively, from retting (−) to (+). ANOVA analyses show that the cellulose (*p*-value = 8.36 × 10^−4^), hemicellulose (*p*-value = 3.22 × 10^−2^), and soluble fractions (*p*-value = 1.45 × 10^−4^) varies significantly. This trend particularly acknowledges that, during the retting stage, microorganisms degrade the soluble fraction of the fibers, which indirectly increases the cellulose content. Similar findings were already observed by Mazian et al., 2018, for hemp fibers [15]. De Prez et al., 2019, observed an enhancement in cellulose content from 64% ± 2% to 72% ± 2% after a dew retting, with a contrariwise decrease of hemicellulose content [6]. However, Martin et al., 2013, observed an enhancement of the hemicellulose content with retting, which they associated with the loss of the soluble content [8].

Figure 3b shows the variation of flax fibers crystallinity index with the retting level. We noticed that the crystallinity index significantly varied from 75.8 for flax (−) to 80.3% for flax (+), with *p*-value of 1.98 × 10^−2^. Zafeiropoulos et al., 2001, obtained a CI of 70.1 and 71.6%, respectively, for flax fibers washed with 5% caustic soda and dew retted [28]. Note that the crystallinity index was a different measure than the percent crystallinity (X_cr_), evaluated by different methods. We considered that the crystallinity index allowed detection of some variation in crystallinity between different characterized samples. Since cellulose is the major contributor to the crystalline fraction of the fiber [17] and the cellulose fraction increased with retting, a direct link can be established between the increase in cellulose fraction and the increase in crystalline index. The observed variation was therefore expected.

### 3.2. Mechanical Analysis of Flax Reinforcements

#### 3.2.1. Technical Flax Reinforcements

Table 3 summarizes the tensile properties of technical flax fibers. The mean value of the results not being appropriate in our case study, we extrapolated them by linear regression to 0 mm and 100 mm to deduce the theoretical values for short (0 mm) and long technical flax fiber (100 mm), respectively. So:

- When the fiber length tends towards 0 mm, the specimen is close to huge continuous elementary fiber. Its mechanical behavior tends to that of elementary fiber.
- When the fiber length reaches 100 mm and over, its mechanical behavior is that of long technical fiber, i.e., a sum of bundles of elementary fibers mainly linked by pectins.

The lower mechanical performances of the 100 mm technical fiber (towards 100 mm) compared to that of theoretical elementary fiber (towards 0 mm) were due to the upper scale of the technical fiber. The latter contained more bundle/bundle interfaces (and fiber/fiber interfaces), which were well known for their weak mechanical properties [29,30]. Moreover, the elastic modulus and tensile strength decreased when the retting level was more pronounced. The ultimate elastic modulus decreased from 96.1 to 60.1 GPa for the theoretical elementary fiber and decreased from 69.9 to 43.6 Gpa for the 100 mm technical fiber. The tensile strength varied from 927 to 809 Mpa and from 702 Mpa to 328 Mpa, respectively, for theoretical elementary fiber and technical fiber (values not presented here). The decrease of the tensile strength and elastic modulus with the increasing of the retting level trend has been observed by Moothoo et al., 2014, for technical fibers [31], but there is no consensus yet. Requile et al., 2018, and Martin et al., 2013, have both shown an improvement of the elementary fiber modulus with retting [8,32]. However, Alix et al., 2012, found no significant impact of retting on the mechanical properties of technical and single fiber [33]

**Figure 4:**
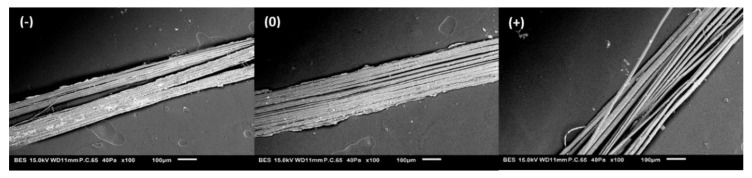
SEM pictures of flax technical fiber retting (−), retting (0), retting (+), (magnification ×100).

By normalizing the mechanical properties by the density, we can observe the same negative impact of retting on the specific mechanical properties of flax fiber (Table 3). The specific elastic modulus decreases from 65.4 to 41.1 GPa·g·cm^−3^ for the theoretical elementary fiber and decreases from 47.6 to 29.8 GPa·g·cm^−3^ for the 100 mm technical fiber. Similar results have been reported by Zhu et al., 2013 [4]. Considering the biochemical and density results, the decrease in mechanical properties could be associated with the biochemical alteration of the technical fibers (loss of soluble compounds, i.e., degradation of middle lamella), which leads to the individualization of the elementary fibers (Figure 4), and consequently reducing the strength and rigidity of the technical fibers.

#### 3.2.2. Flax Bands

**Figure 5:**
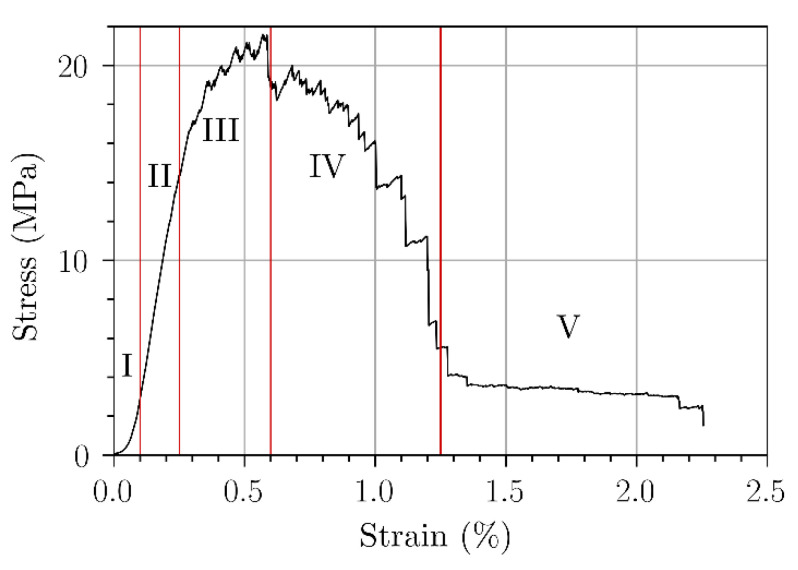
Typical stress–strain curves of flax bands.

Figure 5 shows typical stress–strain curve of flax bands. The typical behavior is similar to untwisted flax fibers, known by the non-linearity of the curve [31]. From the tensile graph, we identified several areas:

- The 1st area (between 0 and 0.1% of elongation) consists of a non-linear stress rise corresponding to the mechanical tensioning of the flax band. The band flax fibers align themselves in the direction of the tension.
- The 2nd area (between 0.1 and 0.3% of elongation) corresponds to a quasi-linear progression of the elastic behavior.
- The 3rd area (starting at approximately 0.3% of elongation) is characterized by a sudden decrease of the stress-strain curve and is associated with a sequential breaking of the fibers as reported elsewhere [14,30].
- The 4th area is where the stress decreases due to decohesion phenomena.
- The 5th area of quasi-constant stress and a weak fibers friction.

**Figure 6:**
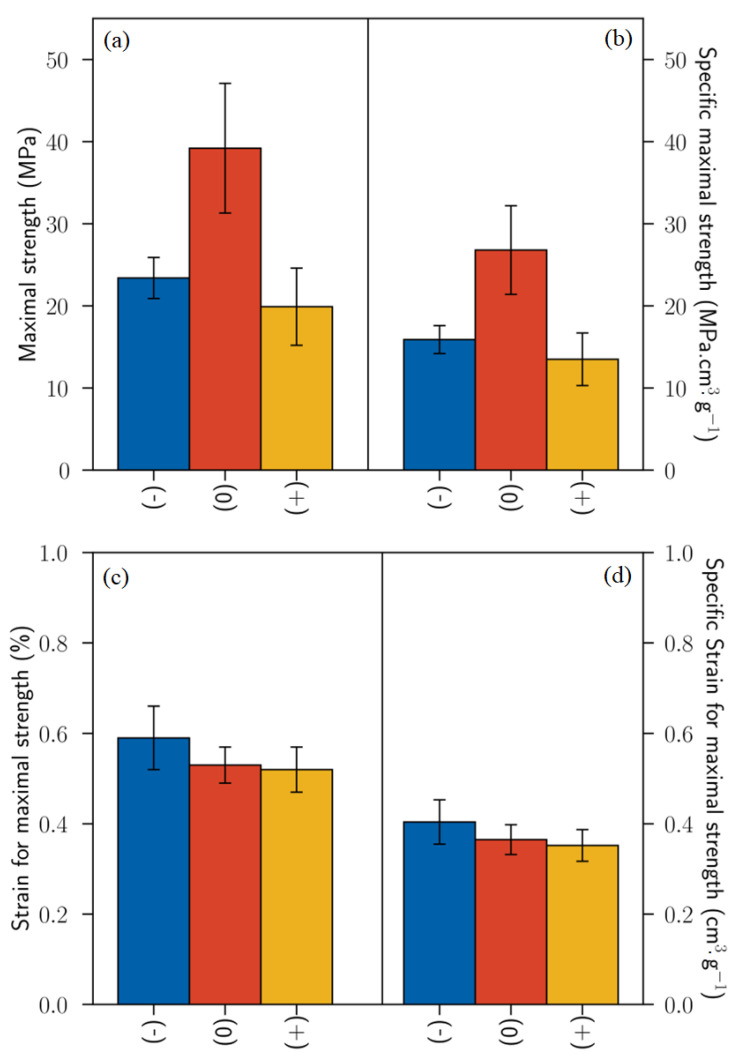
Tensile tests for scutched flax band fibers versus the retting applied (**a**) Maximal strength, (**b**) Specific maximal strength, (**c**) Strain for maximal strength, (**d**) Specific strain for maximal strength. Error bars represent the standard deviation.

The maximum stress strength ranges from 19.9 ± 4.7 MPa to 39.2 ± 7.9 MPa. The retting level (0) seems to be optimal regarding the maximal strength values, as mentioned in Figure 6a. It is interesting to note that the specific mechanical properties of flax bands are very weak and depend on their mass per unit area, according to Khalfallah et al., 2014 [34]. Comparing the results of flax band and technical fibers, we can notice that the maximum stress is much lower in the band scale than in the fibers scale by a factor of 20, on average. This strong difference comes from the intrinsic mechanical properties of the material. At the technical fiber scale, the mechanical properties are mainly dictated by natural interfaces between bundles of fibers and between elementary fibers inside bundles. At the band scale, the mechanical properties are mainly dictated by natural interfaces between technical fibers and between bundles of fibers inside technical fibers. This scale effect logically induces weaker mechanical properties due to poor interface properties.

#### 3.2.3. Bio-Based Composites

Table 4 presents the E1 modulus (calculated between 0 and 0.1% elongation), E2 modulus (calculated between 0.3 and 0.5% elongation), and the maximum stress. ẟE was the loss percentage of the mechanical modulus between E1 and E2. It has been shown that the E1 modulus increased with retting for all the processes used. However, the effect of the retting on E2 and σ was not clear [35]. The best E1, E2, and tensile strength were observed for Setup 3. Additionally, Setup 3 had the highest densities, which was associated with lower internal porosity in the specimens obtained with this process. More details have been given in our previous paper [13].

**Figure 7:**
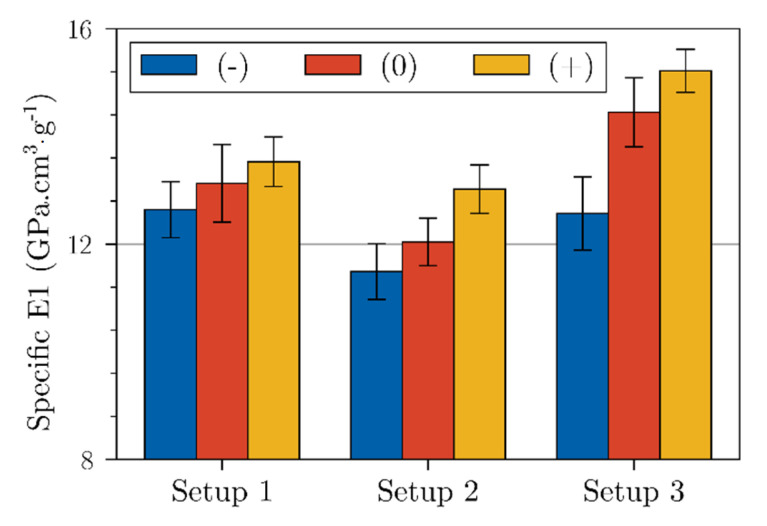
Specific E1 modulus of flax bio-based composites (Error bars represent the standard deviation).

Figure 7 shows specific E1 modulus of our bio-based materials. We observed a positive effect of retting on the specific tensile modulus, the lowest moduli were obtained for materials made with the Setup 2 and the highest ones obtained with the Setup 3 (+). We should note that the density of flax-based materials was impacted by the processing (*p*-value = 1.80 × 10^−2^). Setup 3 provided the densest bio-based materials with an average of 1.29 g.cm^−3^. As indicated in Table 4, the density increasing could be associated with the higher processing temperature, favoring the fiber content and decreasing the internal porosity content [13]. Both the combined effects of retting and the Setup 3 parameters led to a higher tensile modulus, related to the fibers’ individualization (cleaner fiber surface) which favors a better interfacial adhesion with matrix. Moreover, the higher crystallinity rate of the retted fiber also contributed to this mechanical response.

### 3.3. Dynamic Mechanical Analysis

DMA provides pertinent information about the viscoelastic behavior in the solid state. Moreover, it allows the determination of flax composites homogeneity by performing Cole–Cole diagrams which relies on the graphical plotting of the imaginary part (E″) versus the real part (E′), as reported by Devi et al., 2010, and Asim et al., 2018 [36,37]. The authors mentioned that a perfect homogeneous composite shows semicircle Cole–Cole curves.

**Figure 8:**
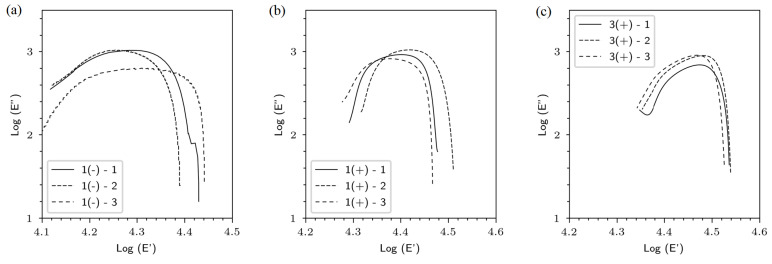
Cole-Cole diagrams of (**a**) 1(−), (**b**) 1(+) and (**c**) 3(+) bio-based materials.

Figure 8 shows the Cole–Cole diagrams plotted for flax bio-based materials. Whatever the tested materials, the curves obtained show a semicircular shape and not perfectly regular, which lets us suspect partially homogeneous composites [36,37]. However, we highlighted that the bio-based composites 1(+) and 3(+) showed a more uniform and regular curves. This may be the result of improved internal homogeneity due to a higher degree of retting (which gave a cleaner fiber surface and a better fiber-matrix interface) and a reduced porosity rate, as shown in our previous work [13]. 1(+) and 3(+) bio-based composites showed the lowest porosity rate of approximately 5%. We noticed also that there was a strong relationship between the internal homogeneity (microscopic scale) and the elastic and viscoelastic performance of the bio-based materials (macroscopic–mesoscopic scale) also reported by Martin et al., 2013.

**Figure 9:**
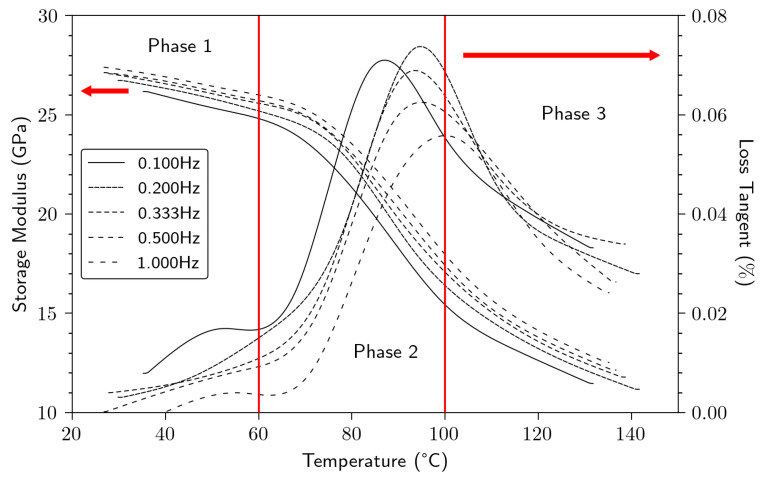
Viscoelastic properties of flax bio-based composite in function of temperature and frequencies for setup 3 (+).

Figure 9 shows typical dynamic mechanical results for bio-based composites and reveals mainly 3 areas:

- Phase 1, when T < 60 °C, characterized by a dominant elastic behavior (E′ > E″). Moreover, we notice a slight loss of the E′ modulus with the temperature increase. It is a typical behavior of a thermoset composite in a glassy state [38,39].
- Phase 2, when 60 < T < 100 °C, was characterized by a sharp drop in the elastic behavior and a strong increase in the viscous part, attributed to a relaxation process, attributed to the glass relaxation of epoxy.
- Phase 3, when T > 100 °C, was characterized by a dominant viscous behavior (E″ > E′), a typical behavior of a thermoset composite in a rubbery state. Both moduli (E″ and E′) continued to decrease significantly.

At mild temperatures (40 °C, for example) and a frequency of 1 Hz, we obtained a storage modulus E′ between 27 to 33 GPa, similar values to those obtained by Duc et al., 2014 [38]. Qi et al., 2023, found a modulus of 2.5 GPa for the neat epoxy at room temperature and at 1 Hz [39], demonstrating the key role of flax reinforcement in terms of stress transfer and elastic behavior [24,38,40]. As the temperature increased, the epoxy softening was more pronounced and the E′ modulus continued to decrease up to 140 °C. The α–relaxation associated with the glass transition phase was clearly visible in the range of 80–100 °C.

Table 5 shows E′ and Tan δ results in more detail. Regardless of temperature and frequency, we noticed a double positive effect of retting and processing parameters on the storage modulus. At mild conditions (40 °C, 1 Hz), E’ increased by 13% (*p*-value = 2.70 × 10^−2^) from Setup 1 to Setup 3. Furthermore, E′ increased by 15% (*p*-value = 1.60 × 10^−2^) from lowest retted (−) to highest retted level (+). This double positive impact was even more pronounced in the rubbery state, where a 45% increase of E′ was observed between retting (−) and (+). A similar trend was also observed by Yang et al., 2014, using kenaf fibers mixed with PBAT-PHBV copolymer matrix composite [41]. As has been highlighted for the static mechanical results, we also noticed the positive impact of retting on the viscoelastic properties. These findings could be associated with fiber individualization, and a cleaner contact surface, allowing both better adhesion and stress transfer.

Regarding the Tan δ results, increasing frequencies induced a shift of the glass transition to a higher temperature (from 81 to 100 °C), also observed by Pillai et al., 2016 [42]. We can note that the highest glass transition is obtained for the setup 1 (−). However, the lowest is obtained for setup 3 (+), attributable to a less cross-linked system allowing a higher chain mobility in a less constrained system.

**Figure 10:**
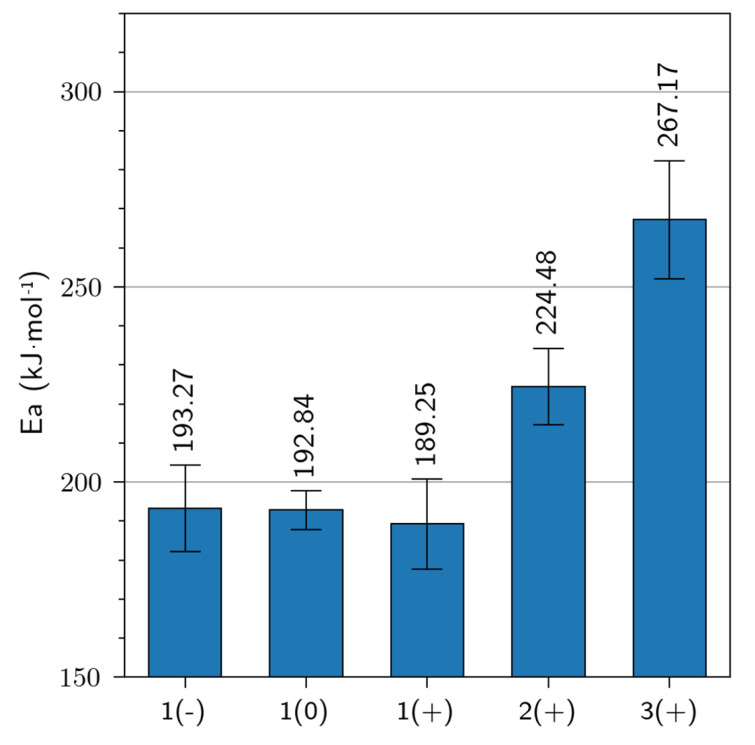
Activation energy of glass transition by DMA analysis (Error bars represent the standard deviation).

To better understand the processing influence on the glass transition, Figure 10 shows the activation energy (Ea) of the glass transition of all samples. To determinate the activation energy we applied an Arrhenius fit on the temperature peak for all frequencies used. The Arrhenius equation is given in Equation (3):

(3) $$f=f_{0}e^{\left(-\frac{E_{a}}{RT}\right)}$$

where f being the measuring frequency, $f_{0}\;$is the frequency when *T* approach infinity, *R* is the gas constant (8.31446262 J.K^−1^·mol^−1^) and *T* is the temperature of the tan δ peak [43]. The activation energy ($E_{a}$) of the glass transitions ranged from 189 to 267 kJ·mol^−1^. It appears that retting had no significant impact on the activation energy (*p*-value = 0.9), but a processing effect was very pronounced (*p*-value = 1.80 × 10^−2^). This was related to the curing efficiency, estimated at 96.7, 93.6, and 91.7% for bio-based composites made with setup 1, 2, and 3, respectively (more detail in our previous paper). According to Wu et al., 2018, at a curing rate > 85% the mobility of the molecular chains was progressively restricted due to the development of the reticulated structure, which progressively increased the required energy for the glass transition [44]. It greatly restricted the cooperative motions and configuration rearrangements, requiring high activation energy values.

## 4. Conclusions

The flax retting and the processing parameters governed the mechanical properties of flax-epoxy bio-based composites materials at different scales (technical flax fiber, fiber band, and bio-based materials). At the scale of flax technical fiber, a biochemical alteration of the technical fiber was highlighted as the retting level increased and was associated with the degradation of the middle lamella, which favored the individualization of the flax fibers. A direct link was established between the biochemical alteration of technical flax fibers and their specific mechanical properties. The degradation of the middle lamella, mostly made of pectin, explained the loss of elastic properties due to retting. The crystallinity index also seems to increase with retting, which was an expected result as retting positively affected the cellulose content. Furthermore, the mechanical properties of flax technical fibers were better than the flax band. For the last, the weaker interface quality was the responsible of these poor properties.

At the bio-based composites scale, Setup 3 (T = 160 °C) and the high retting level (+) were the most relevant for a better mechanical response of flax bio-based materials. The same trend was also deduced for viscoelastic behavior. With optimal conditions (retting (+) and improved processing Setup 3), the storage modulus was also significantly improved, confirming the efficiency of retting (+)/Setup 3 conditions at different scales of mechanical performances. In these conditions, fiber individualization was more pronounced, and a better fiber-matrix interface can be achieved. We also highlighted that the α-relaxation and the activation energy ($E_{a}$) were mainly affected by the temperature process. It will be necessary to focus on the interfacial analysis and wettability mechanism for flax/epoxy system.

## Figures and Tables

**Table 1:** Flax cultivation conditions.

	Seeding	Harvesting	Winding (W)	Total Precipitation (mm)
Under retted (−)	April 10th, 2017	July 13th, 2017	W1 = August 7th, 2017	17.2
Nominally retted (0)			W2 = August 28th, 2017	66
Over retted (+)			W3 = September 22nd, 2017	130

**Table 2:** Bio-based composites processing program.

	Retting	t (min)	T (°C)	P (Bars)
1	(−)	370	140	50
	(0)	370	140	50
	(+)	370	140	50
2	(−)	175	140	25
	(0)	175	140	25
	(+)	175	140	25
3	(−)	130	160	50
	(0)	130	160	50
	(+)	130	160	50

**Table 3:** Tensile tests for flax fibers.

Modalities		Ultimate Elastic Modulus (GPa·cm^3^·g^−1^)	Ultimate Tensile Strength (MPa·cm^3^·g^−1^)	Specific Ultimate Elastic Modulus (GPa·cm^3^·g^−1^)	Specific Ultimate Tensile Strength (MPa·cm^3^·g^−1^)	Density (g·cm^−3^)	Ultimate Failure Strain (%)
Extrapolations to 0 mm length	(−)	96.1 ± 2.6	927 ± 25	65.4 ± 1.8	631 ± 17	1.470 ± 0.04	0.84
	(0)	85.8 ± 0.6	897 ± 6	58.4 ± 0.4	612 ± 4	1.464 ± 0.01	0.97
	(+)	60.1 ± 0.8	809 ± 11	41.0 ± 0.6	553 ± 8	1.463 ± 0.02	1.09
Extrapolations to 100 mm length	(−)	69.9 ± 1.9	702 ± 18	47.5 ± 1.3	477 ± 12	1.470 ± 0.04	0.79
	(0)	59.1 ± 0.4	513 ± 3	40.4 ± 0.3	350 ± 2	1.464 ± 0.01	0.75
	(+)	43.6 ± 0.6	328 ± 1	29.8 ± 0.4	224 ± 3	1.463 ± 0.02	0.69

**Table 4:** Mechanical results for bio-based composites.

Bio-Based Composites	E 1 (GPa)	E 2 (GPa)	σ (MPa)	ẟE (%)	Density (g·cm^−3^)
1 (−)	15 ± 1.91	12 ± 1.02	287 ± 25.3	20.17 ± 2.78	1.220 ± 0.015
1 (0)	16 ± 0.98	12 ± 0.74	322 ± 23.1	23.59 ± 1.86	1.253 ± 0.010
1 (+)	17 ± 0.96	12 ± 0.76	279 ± 17.9	27.58 ± 3.24	1.262 ± 0.009
2 (−)	14 ± 0.58	11 ± 0.42	283 ± 14.5	22.25 ± 1.16	1.244 ± 0.006
2 (0)	15 ± 1.69	11 ± 0.28	323 ± 33.9	21.21 ± 1.92	1.253 ± 0.010
2 (+)	16 ± 1.46	11 ± 1.23	290 ± 36.1	27.78 ± 3.58	1.258 ± 0.009
3 (−)	16 ± 0.83	12 ± 0.65	250 ± 16.2	25.28 ± 1.76	1.297 ± 0.004
3 (0)	18 ± 0.57	14 ± 0.57	350 ± 17.4	23.98 ± 1.13	1.293 ± 0.018
3 (+)	19 ± 0.91	13 ± 0.59	381 ± 32.1	31.11 ± 1.88	1.280 ± 0.016

**Table 5:** DMA results.

Bio-Based Composites	0.1 H			1 Hz		
	E′ (40 °C)	E′ (30 °C)	Tg (Tan δ)	E′ (40 °C)	E′ (130 °C)	Tg (Tan δ)
1 (−)	26.05 ± 0.90	11.53 ± 0.12	87.93 ± 4.47	26.94 ± 0.83	12.91 ± 0.29	100.60 ± 1.40
1 (0)	26.30 ± 0.64	15.67 ± 0.13	81.93 ± 1.27	27.26 ± 0.67	17.09 ± 0.07	93.37 ± 3.45
1 (+)	29.97 ± 1.61	18.26 ± 0.64	82.93 ± 5.05	31.71 ± 2.04	18.26 ± 0.64	82.93 ± 5.05
2 (+)	29.04 ± 1.74	19.22 ± 0.20	86.20 ± 5.88	30.19 ± 1.98	20.60 ± 0.40	96.33 ± 4.57
3 (+)	33.75 ± 0.49	21.33 ± 0.46	81.87 ± 1.51	34.99 ± 0.53	23.13 ± 0.34	89.37 ± 0.15
